# Thermal Conductivity and Temperature Dependency of Magnetorheological Fluids and Application Systems—A Chronological Review

**DOI:** 10.3390/mi14112096

**Published:** 2023-11-13

**Authors:** Seung-Bok Choi

**Affiliations:** 1 Department of Mechanical Engineering, The State University of New York, Korea (SUNY Korea), Incheon 21985, Republic of Korea; seungbok.choi@sunykorea.ac.kr; 2 Department of Mechanical Engineering, Industrial University of Ho Chi Minh City (IUH), Ho Chi Minh City 70000, Vietnam

**Keywords:** magnetorheological fluid, temperature effect, thermal conductivity, field-dependent properties, heat transfer

## Abstract

Many studies on magnetorheological fluid (MRF) have been carried out over the last three decades, highlighting several salient advantages, such as a fast phase change, easy control of the yield stress, and so forth. In particular, several review articles of MRF technology have been reported over the last two decades, summarizing the development of MRFs and their applications. As specific examples, review articles have been published that include the optimization of the particles and carrier liquid to achieve minimum off-state viscosity and maximum yield stress at on-state, the formulation of many constitutive models including the Casson model and the Herschel–Bulkley (H–B) model, sedimentation enhancement using additives and nanosized particles, many types of dampers for automotive suspension and civil structures, medical and rehabilitation devices, MRF polishing technology, the methods of magnetic circuit design, and the synthesis of various controllers. More recently, the effect of the temperature and thermal conductivity on the properties of MRFs and application systems are actively being investigated by several works. However, there is no review article on this issue so far, despite the fact that the thermal problem is one of the most crucial factors to be seriously considered for the development of advanced MRFs and commercial products of application systems. In this work, studies on the thermal conductivity and temperature in MRFs themselves and their temperature-dependent application systems are reviewed, respectively, and principal results are summarized, emphasizing the following: how to reduce the temperature effect on the field-dependent properties of MRFs and how to design an application system that minimizes the thermal effect. It is noted here that the review summary is organized in a chronological format using tables.

## 1. Introduction

Since Rabinow discovered a new type of magnetic fluid at the National Bureau of Standards in 1948 [1], numerous works on the development of advanced magnetorheological fluids (MRFs) and semi-active control systems, utilizing them as a vehicle suspension damper, have been actively carried out since the early 1990s. In Rabinow’s work, many research issues arose that required serious consideration to develop a high control performance of the proposed clutch. These issues include the saturation of the solid phase under the magnetic field, an optimal mixture between the particles and the carrier liquid to avoid the de-energized problem, sealing to protect from liquid leakage, the design of an appropriate magnetic circuit, power consumption, a wear problem and a lubrication issue, which is different from a dry friction clutch. Therefore, many scholars, who are actively working in the rheology-related fields ofchemistry and mechanical engineering associated with control logics, have tried to solve the above issues. As a consequence of this great effort and long-term research, MRFs are commercialized by Lord Company [2,3]. They sell several different MRFs, named MRF-122EG, MRF-132DG, and MRF-140CG, as well as several application systems, such as the MR damper. On the homepage of Lord Company, there are many questions and answers regarding the commercial MRF products, such as: (i) how does the iron affect the properties of MRF? (ii) How is MRF viscosity measured? (iii) What determines the viscosity of MRF? (iv) What is the best model for how MRFs behave? (v) What is the density of MRF? (vi) What is the molecular weight of MRF? (vii) What kind of iron particles are used in making Lord MRF? (viii) Does the MRF change volume during activation? (ix) How does fluid settling affect the performance of MRF devices? (x) Are iron particles in oil abrasive? (xi) What is the time response of MRF? (xii) How do temperature and thermal conductivity affect MRF? So far, numerous works on the above issues have been undertaken, to resolve these problems and hence make more advanced MRFs and applications. Since there are many works on the same or similar research topics, several review reports on the above-mentioned studies have been published in the last 20 years, the topics of which include: the development of advanced MRFs, a review of sedimentation stability, a review of the constitutive models of MRFs, a review of the role played by particle shape and morphology in MRFs, a review of challenges and solutions in the preparation of MRFs, a review of abrasive finishing using MR fluids, and a review of the physical mechanisms and microchemical models of MRFs. On the other hand, as for the development of MRF application systems, many review articles have been published in the last two decades, including the types of MR damper, medical and rehabilitation applications, the field-dependent characteristics of clutches and brakes, self-powered MR dampers, vibration control for automotive suspension systems and civil structures, sensor applications, and the types of mount systems for impact mitigation. 

It is obvious from the above literature survey of review articles, that a review article focusing on the last question (xii) made by Lord Company has not been reported, as far as the author’s knowledge is concerned, even though the amount of research on this topic is gradually increasing these days. In answer to this question, the company catalog states that the temperature effect at the off-state is largely dependent on the on-state magnetic coil and the working conditions. However, there is no accurate information about the properties of MRFs at lower temperatures ranging from 0 to 40 °C. Moreover, there is no detail on how to mitigate the temperature effect or how to design an application system that minimizes the thermal effect and hence prevents performance degradation. Consequently, recent works regarding the effect of temperature on MRF itself and its applications need to be summarized. In this work, firstly, major review articles on the development of enhanced MRFs and some review articles on the practical development of MRF application systems are revisited. Subsequently, the effects of temperature and thermal conductivity on the properties of MRFs, and the thermal effect on the performances of application systems are reviewed in detail and summarized in chronological order using tables. Since the tables provide the main information regarding the issue of temperature, readers can easily understand the related important research history and can gain several creative insights regarding some challenging issues to be resolved in future. 

It is noted here that Section 2 and Section 3 have been devoted to review articles on MRFs and their application systems in a chronological manner. These review articles have been written in several different formats dependent on the theme and the time of publication. For example, one review article on MRF focused on the trade-off between sedimentation stability and MR effect, with respect to different additives or/and coating thicknesses, while one review article discussed many different types of MR dampers, stating the advantages and disadvantages of each type of MR damper. Therefore, it is hard to understand the research history on MRFs and their application systems. Hence, these review articles are revisited and formatted in a chronological order, to easily identify the developing history of MRF technology year by year. The fundamental backgrounds of the field-dependent characteristics of MRFs and the application systems studied so far can help the reader to understand the temperature problems that are reviewed and discussed in this review work.

## 2. Review Articles on Magnetorheological Fluids

Carlson [4] wrote a review article introducing commercial magnetorheological fluids (MRFs), made by Lord Company, stating a series of figures of merit. It introduced commercial MRFs and the first MRF application device, which was a small controllable brake applied to aerobic exercise equipment. In 1998, a small MR seat damper was commercialized for heavy-duty trucks, followed by several types of MR dampers for the suspension systems of race and sedan vehicles. In this review article, the ingredients of the MRFs developed by Lord Corporation in 2004 and their field-dependent rheological properties are also presented. For example, an MRF consisting of a hydrocarbon carrier liquid and an iron particle volume fraction of 22% can produce a maximum yield stress of 23 kPa at 200 kA/m and a response time of less than 1 ms. Several advantages of semi-active controllable actuators, introduced in this review article, significantly triggered numerous research works, in diverse areas with a creative design philosophy, which replaced traditional systems or devices with high performance systems showing design simplicity, a low power consumption, and an excellent adaptability to various control strategies. Muhammad et al. [5] summarized several requirements for making high-performing MRFs, by stating principal properties including the lowest coercivity, the highest saturation magnetization, the fastest response time, non-abrasive particles, spherical-shaped particles, and high purity particles (carbonyl iron powder: CIP). In addition, the particle volume fraction and particle size dependence of the viscosity was described, explaining that a higher volume fraction, led to a higher viscosity, but also to a faster sedimentation due to the increased density. Some constitutive models of MRFs, relating to the shear stress versus the shear rate, were also given by classifying its geometry into concentric cylinder, parallel plate, cone and plate, and double concentric models. Ierardi and Bombard [6] introduced the optimized mixture method for CIPs and a carrier liquid (hydrocarbon) to achieve minimal off-state viscosity, as well as maximal field-dependent yield stress. In their investigation, three different BSAF CIPs were been used to obtain the proposed target, because the CIPs had several merits over other magnetic particles, such as excellent absorption of radar and microwaves, high purity, outstanding quality and consistency, reliable delivery, compatibility with most polymer matrices, and being easily compoundable. Therefore, CIPs are currently used to develop innovative solutions for a whole spectrum of different applications, including automotives, since BASF discovered the CIP processing recipe 85 years ago. Three samples were classified into Sample A (coarse), Sample B (medium), and Sample C (fine), and dispersing thixotropic additives were used in all mixture formulations. It was found in this investigation that Sample A produces the highest field-dependent magnetization effect, indicating that a larger particle size contributes to a high yield stress. de Vicente [7] comprehensively revisited the most salient properties of the field-dependent rheological fluids of MRFs, especially focusing on researchers’ understanding of flow motion, yield stress, and viscoelastic behavior under shear mode operation. Firstly, they reviewed the manufacturing methods of MRFs via traditional approaches, followed by new methods aimed at reducing particle sedimentation by reducing the particle size to the nanometer range. Then, they discussed the particle magnetization model (a constitutive model of MR fluids) from both a microscopic and macroscopic perspective, by using the Brownian motion, Mason number, Reynolds number, Peclet number, van der Waals body forces, Bingham model, Herschel–Bulkley model, and the Casson method. Subsequently, the relationship between the shear stress and shear rate, and between the viscosity and the Mason number were reviewed, at various magnetic field intensities, to understand the constitutive models of MRFs. Ashtiani et al. [8] reviewed the overall content of MRFs, especially emphasizing the different methods of preparation and stabilization, the field-dependent rheological models, and the applications. The MR effect was found to be characterized by a reversible increase in the viscosity and the yield stress, due to the introduction of a magnetic field, which can be explained by the particle chain formation. The MR effect could be controlled by the magnetic field’s intensity and the rheological characteristics of the MR fluid constituents. This review article pointed out the difficulty of practical usages, due to the particle sedimentation caused by the density mismatch between the magnetic particles and the carrier liquids. As a solution to mitigate this problem, several approaches were suggested: particle coating, the use of various additives, the use of various carrier liquids, the use of porose particles, the use of nano-sized particles, the use of different particle shapes and so forth. A detailed schematic image of the MR effect were shown, both with and without adding nanoparticles to MRF dispersed in the voids between microparticles, resulting in yield stress by strengthening the particle chains. The significance of the carrier liquid was also discussed, and one potential carrier liquid was suggested: polyethylene oxide (PEO), which is a widely used polymer since it is a linear polymer soluble in organic media. Other potential carrier liquids included hydro-carbonic oil, ferrofluid, silicone oil, mineral oil, ionic liquid, cedar wood oil, and so on. 

Choi [9] comprehensively reviewed and analyzed the methods to improve sedimentation stability by altering the aspects of three ingredients: particle modification, carrier liquid modification, and the adjustment of additives. In addition, a few conceptual methodologies to prevent the sedimentation that occurs during the bottle’s storage and in the application systems were also discussed as possible obstacles to developing successful MR applications. For the particle modifications, several types of magnetic particles, including CIP, iron oxide, iron carbide, low carbon steel, silicone steel, nickel, and cobalt, were used and coated particles were employed; as for the carrier liquid, many types of carrier liquids aimed at reducing the density mismatch, such as mineral oil, modified silicone oil, hydrocarbon oil, polyalphaolefin (PAO), 1-ethyl-3-methylimidazolium diethyl phosphate, and 1-hexyl-3-methylimidazolium chloride, were investigated. The additives also play a crucial role in the sedimentation stability of MRF and, hence, the effect of using additives, such as thixotropic agent, carboxylate soap, antioxidant, lubricant, viscosity modifier, metal oxide powders, sulfur-containing additives, thioesters, xanthan gum, and stearate carboxylic acid, on MRF sedimentation were discussed. The review article [10] also analyzed the sedimentation stability of MRFs, focusing on the use of different additives: iron oxide additives, ferrofluids, organic additives, carbon allotropes, and inorganic additives. In addition, the relationship between the carrier liquid and sedimentation was investigated by using several different carrier liquids: linear polydimethylsiloxane, hyperbranched polycarbosilane, CI dispersed in silicone oil (Si), synthetic oil (Sy), sunflower oil, and polytetrafluoroethylene (PTFE). Pei and Peng [11] summarized the diverse constitutive models of MRFs, which are useful tools for the prediction and analysis of MRFs’ field-dependent characteristics, such as yield shear stress and complex modulus. After describing the constituents and rheological properties of MRFs, two types of constitutive modeling methods were discussed: macroscopic models and microscopic models. The macroscopic models, which are generally data-based models, have been widely used due to their simplicity and accuracy. Among the many macroscopic parametric models, the Bingham model is the simplest, but provides useful properties, including the shear stress versus shear rate at different magnetic field intensities. The macroscopic parameter models include the biplastic model, Casson model, biviscous model, Herschel–Bulkley (H–B) model, Eyring model, Robertson model, Pa–Casson model, Mizrahi–Berk (M–B) model and so forth. The prediction of the accuracy of these models is dependent on many factors such as particle type and size, temperature, types of carrier fluids, and the properties of the additives. In general, the parametric models lose their prediction accuracy at both very high and low shear rates. Thus, there are several types of macroscopic nonparametric models, which can be applied for the prediction of the field-dependent shear stress of MRFs which are subjected to uncertainties of varying magnetic fields and varying currents. Unlike the parametric models, nonparametric models have difficulty expressing specific equations containing experimental coefficients. One of the best ways to deal with this nonparametric problem is to use the artificial neural network (ANN) and support vector regression (SVR) techniques. Using these methods, the time-varying data are trained and tested by defining the temperature and shear rate as the inputs and the shear stress as the output. Another approach to resolve the uncertainties is to use an extreme learning machine, which can demonstrate the properties of both the ANN and SVR with a smaller training time. Additionally, the nonlinearity and saturation of particle magnetization and the calculation of the resistance force of the chains in the field-on microscopic models have been studied by several scholars. Some representative microscopic models include the finite element model, micro–macro model, dipole model-based micro–macro model, initial tilt chain model, and the micro model based on a hexagonal closed packed structure. 

Kumar et al. [12] summarized a few challenging methods for the development of advanced MRFs, considering the types of magnetic particles obtained by the chemical vapor deposition of iron pentacarbonyl, and the particle shapes that affect the wear on the walls of the container or device inside in which MRFs operate. To increase the sedimentation stability of MRFs, several methods were discussed: the use of coated particles and the use of additives such as carboxymethyl cellulose, polyethylene oxide, polyvinyl butyral, fibrous carbon, oleic acid, cholesteryl chloroformate, and magnetic nanoparticles. When the additives were used to reduce the sedimentation, the percentage of the volume or weight needed to be carefully adjusted. In addition, the fast time responses of MRFs, dependent on the input current and the friction between the particles in fluid flow, were carefully investigated. Matharu and Sehgal [13] summarized the field-dependent rheological MRFs focusing on the particle type, particle shape, and particle size, because these factors significantly influence the yield shear stress, sedimentation stability, wear and durability, and the magnetic intensity. Firstly, MRFs were classified into monodispersed, bi-dispersed, poly-dispersed, ferrofluid-based and dimorphic fluid-based, stating inherent characteristics such as sedimentation and off-state viscosity. Particulate materials included carbonyl iron, Fe_3_O_4_, cobalt, nickel, carbon nano tube, graphene oxide, glass, hard magnetic metal, soft magnetic metal, magnetic stainless steel, and alloy iron. The size of the particulates ranged from nanometers to 30–50 μm. The durability of MRFs caused several disadvantages, such as degradation due to the oxidation of the particles, degradation due to the wear of the particles, degradation due to breakage or spalling of the particles, and the decrease in loss modulus over time due to shear thinning. On the other hand, Khairi et al. [14] reviewed numerous studies that focused on the ability of these materials to alter their rheological properties in response to applied magnetic fields. In particular, in this review, the influence of additives on the rheological properties of MR solids, including MR elastomers and MR greases, were presented. It was shown that plasticizers soften the rubber matrix, increasing the MR effect by lowering the zero-field moduli, and carbon-based additives provide superior bonding with the rubber matrix and improve the dispersion of CIPs at the same time. Chromium-based additives enhance the stability of CIPs in the dispersion media by acting as coating agents, resulting in the prevention of agglomeration. It was also demonstrated that a combination of plasticizer, multi-walled carbon nanotube (MWCNT), and carbon black used during the fabrication of anisotropic MR elastomers enhance the MR effect. Notably, MWCNTs provide an enhancement of MR elastomers, MR gels, and MR plastomers. It was noted that, to obtain better additives to improve the MR effect in various aspects, mathematical relationships must be established to predict the best composition of additives, with systematic investigations on the interparticle forces between particles and additives, as well as additives and matrices.

Table 1 presents several review articles related to temperature and the thermal conductivity of MRFs. As seen from the table, MRF products were on the market between 2000 and 2009 showing high yield stress at the on-state and low viscosity at the off-state; several approaches to enhance the temperature robustness of MRFs were proposed between 2010 and 2019; and works to reduce the particle sedimentation, and to investigate constitutive models and the effects of several additives on the field-dependent properties of MRFs were undertaken after 2020.

## 3. Review Articles for MRF Applications 

As mentioned in the Introduction, various types of systems and devices have been proposed and developed that utilize MRFs. The most significant MRF technology has been highlighted by the MR damper, for use in a semi-active suspension system in the automotive engineering field. Therefore, review articles considering vehicle shock absorbers (dampers) have been written by many scholars. Imaduddin et al. [15] reviewed rotary MR dampers instead of traditional linear MR dampers, focusing on several merits of the rotary type including its design compactness, reduced weight, and reduced cost, due to the lack of pressure chambers filled with MRF, hence the low requirement for MRFs. However, since the field-dependent yield shear stress should be produced by rotational motion, the structural configurations of the rotary MR damper are relatively complex. The rotational applications such as MR clutch, MR brake, and hybrid types of MR brakes and clutches are relatively easy to make, by just installing the magnetic coils in an appropriate place. The authors also investigated the modeling methods for rotary MR dampers: the Bingham-based torque model, the Herschel–Bulkley-based torque model, the Bouc–Wen model, and the Dahl model. It was remarked, at the end of the discussion, that an accurate modeling of the hysteresis model of rotary MR damper, an innovative application adaptable to the rotary damper, an accurate formulation of the constitutive model, and the design of an appropriate controller with high stability and robustness under severe uncertain operating conditions, are challenging issues for future works to resolve to enable the successful development of rotary MR dampers. Kaluvan et al. [16] summarized the sensor applications of MRFs, instead of the actuator applications covered by numerous scholars. In particular, the design and principle of the following novel sensors were briefly introduced: resonant sensors, current sensors, magnetic flux sensors, and tactile sensors. This article introduced a novel electrical current measurement technique using MRF in its shear mode of operation, and tactile displays for a robotic system were discussed, which would be applicable to minimally invasive surgery (MIS), to provide a surgeon with tactile information about remote biological tissues or organs. Do and Choi [17] comprehensively reviewed the design configurations of a high load MR mount, which was applicable to construction vehicles and ship engine mounts, for a reduction in unwanted vibrations or impact loads. It was firstly summarized in the design review that, in the design process of high loaded mounts, many principal factors, such as damping gaps, cross sections, and the equations of damping forces, should be critically considered. In addition, identification models have to be carried out simultaneously, to achieve a superior vibration control performance. It was found that the high loaded MR mount is designed based on two main modes of MR fluid: flow mode and shear mode. The distribution of parts inside the mount determines the establishing pressures, which are directly related to the damping force of the mount. It was also identified that the high loaded MR mount, based on the squeeze mode, is still difficult to design and manufacture due to the low damping force and the leakage of the fluid itself. Hence, a breakthrough design configuration, using the squeeze mode, with large deformation values needs to be created in future. Ahamed et al. [18] reviewed both traditional MR dampers and self-powered MR dampers, considering their structural configurations, governing dynamic models, and energy generation. Various MR applications, including automotive dampers, industry brakes and clutches, glass polishing, hydraulic valves, composite core structures, active servo valves, and rotary seals were investigated and their operation performances were discussed, in terms of design simplicity, power consumption, performance, and leakage issues. Subsequently, several mechanical models of MR damper associated with the governing equations were discussed by adopting the Bingham model, the Bouc–Wen hysteretic operator-based model, the simple Bouc–Wen model, the modified Bouc–Wen model, the hyperbolic tangent function-based model and the nonlinear biviscous model. In addition, energy harvesting (self-powered) MR dampers were introduced, and the structural configuration was described, which is integrated with the electromagnetic induction (EMI) and the permanent magnet in which the EMI system can produce the generated voltage linearly proportional to the relative velocity across the MR damper. Finally, other methods to create self-powered MR dampers, such as the use of induction coils, rack and pinion mechanisms, ball and screw mechanisms, and generators and motors were also briefly summarized. Choi et al. [19] investigated several control strategies which were applied to various devices or systems utilizing MRFs and MREs. The mandatory requirements for successful applications of MRF and MRE include several factors: advanced material properties, optimal mechanisms, suitable physical modeling, and appropriate control schemes. Among these requirements, the use of an appropriate control scheme is a crucial factor, since it is the final action stage of the application systems to achieve desired output responses. There are numerous different control strategies which have been applied to many different application systems of MRF and MRE: skyhook controllers, PID controllers, LQR/LQG (linear quadratic regulator/linear quadratic Gaussian) controllers, sliding mode controllers, adaptive fuzzy controllers, neural network controllers, and hybrid controllers that combine more than two different control schemes. The advantages and disadvantages of each control scheme were discussed so that future researchers could develop more effective control strategies to achieve higher control performances of the many application systems that utilize MR materials.

Kumari and Chak [20] comprehensively surveyed and described utilizing MRFs in surface finishing (or polishing). Magnetically assisted abrasive finishing (MAAF) processes are precision material removal processes that have been applied to a large variety of materials, from brittle to ductile and from magnetic to non-magnetic. As is well known, the MAAF process relies on a unique “smart fluid”, known as magnetorheological (MR) fluid or electrorheological (ER) fluid. Nowadays, MR fluids are more popular for surface finishing, as they contain micron-sized magnetizable particles, such as carbonyl iron, dispersed in a non-magnetic carrier medium like silicone oil, mineral oil, or water. The mechanism involved in MRF-facilitated material removal and surface finish basically comprises three different modes: (i) the abrasive particle is held by chains of iron particles, (ii) the bunch of iron and abrasive particles moves in a forward direction and shears/removes a very small amount of material in the form of micro-chips, and (iii) when this bunch of iron and abrasive particles moves further, it separates the micro-/nano-chip from the work piece. There are several finishing or polishing approaches based on the above three modes: ball and MR finishing, permanent magnetic finishing, chemical-based MRF processing, and ultrasonic based MRF processing. As for effective applications, the machining of electronics and industrial components, the machining of optical components, and the machining of biomedical component were fully discussed, followed by challenges that needed to be solved for successful commercialization. For example, how can the finishing time be reduced when the ball and MR fluid (BMRF) process is applied to hard material? And how can the issue of the MRAFF process being applied to specimens with sharp edges be solved? This review article is very helpful for its knowledge of surface processing that utilizes MR fluids and of creating new methodologies that solve existing disadvantages. Daniel et al. [21] summarized MR dampers, focusing on civil structure applications to suppress unwanted vibrations through passive and semi-active control means. The main difference between MR dampers used for automotive suspension and those used for civil structures is that much higher yield stress (actuating force) is required for the civil structures. Many researchers proposed MR dampers that can generate more than 20 kN at around 3A and can be applied to precision table structures, high story buildings, bridge cables, and seismic structure, to reduce vibrations as quickly as possible. Also, controllers for the semi-active system, skyhook controllers, linear quadratic Gaussian (LQG) controllers, and fuzzy controllers have been applied to vibration control in high story buildings and following seismic occurrences. Several works were also introduced where an optimization method using the genetic algorithm was used, and bang-bang and clipped optimal controllers were implemented to reduce the vertical acceleration. The review article finally remarked that the important factors in developing successful technology in civil structures are fast reaction time, designs with fewer moving parts, and a choice of appropriate control strategies. Sohn et al. [22] briefly reviewed the applications of MRF and ERF in robotic systems. Since ERF has a lower stiffness effect, its applications for flexible robot arms, soft robots, flexible gantry arms, tactile robot displays, and long and thin robot arms such as those used by space robots, have been studied by several scholars. Many studies in the robotic field using MRF have also been actively undertaken. Among many different types, haptic robots, collaborative robots, climbing robots, spherical robots, planar robots, deformable grippers and many different rehabilitation systems, such as knee prosthetics, have been discussed. Advantages of robots fabricated from MRFs include accurate interaction information between the human and the robot by controlling the current, and easy fabrication using a 3D printing method. This review paper can motivate the creativity of researchers seeking to harness MRF for robotic applications. Ahamed et al. [23] reviewed the field-dependent properties of MR materials and comprehensively investigated MR materials-based applications. They summarized the properties of MR materials, including MRFs, MREs, MR foam, MR grease, and MR plastomers, that have been studied in the last two decades. In this review article, the applications were focused in several engineering and technology fields, such as the automotive industry, the civil environment, the military sector, and medical devices. More specifically, they summarized the development procedures of MRF brakes, MRF dampers, MRF polishing, MRF dampers for civil structures, diverse MRF seat dampers, MRF dampers for washing machines, MRF dampers for above-knee amputees, and a human body palpation sensor using MRF. Oh and Choi [24] reviewed various types of MR dampers applicable to different purposes, whose design configurations were discussed. After briefly explaining the salient characteristics of MR fluids, they discussed MR dampers for automotives, MR dampers for bridge cables, MR seat dampers for commercial vehicles such as trucks and buses, MR dampers for washing machines to suppress both vibration and noise, a lateral type of MR damper for high-speed railway vehicles to improve their operating stability at curve lines, and special MR dampers for military vehicles subjected to severe environmental conditions of temperature and road condition, in terms of their current status and directions for future works. In addition, the performances of many different types of MR dampers applicable to human rehabilitation devices, prosthesis, and above-knee amputees were described. In this review article, they also surveyed the development, application, and classification of MR dampers, focusing on the structural components, operating principles, control performances, and the developing trend for various systems and devices to be operated by semi-active control strategies.

Kolekar et al. [25] reviewed the research trend on sandwich structures, whose stiffness and damping properties could be controllable, utilizing MRF as cores or layers. They firstly demonstrated that both the damping property and the stiffness property of the sandwich structure can be effectively controlled in several ways: the change in the field intensity, the location of core zones, the partial and full treatment and boundary conditions of the structures. In addition, it was found that the modal parameters, such as the natural frequency of the sandwich structures, are controllable in real time by supplying an external input current to the specific points, and, hence, the resonance cased from the external excitations can be easily avoided by changing the natural frequencies of the sandwich structures. In addition, the controllability of the mode shapes of the sandwich plates can provide several advantages in flexible structure systems such as aircraft wings, automobile hoods, and the appendages of space robots. Despite many works describing the advantages of these smart sandwich structures, there are no specific practical applications in the real world so far. This directly indicates that further research should be explore the optimal coil structure for MRFs, the maximization of the controllable range of the stiffness and damping properties, the buckling problem as a function of controllable parameters, and the field-dependent acoustic characteristics with controllable cores. Yuan et al. [26] investigated types of MR dampers along with the position of the magnetic circuit core and flow modes. It was found that controlling the viscosity of an MR damper in a limited axial channel can extend the variation of both the damping ranges and force, due to different positions and turns of coils. In addition, by adopting a flow mode MR damper with a radial and circumferential direction, a longer length of damping channels can be obtained. In particular, combining radial and circumferential flow modes in a miniature external valve can provide more excellent performances. As targets for future works, they identified creating MR dampers with a longer damping channel, high magnetic field utilization, larger damping range, weak magnetic field excitation, smaller volume, less energy consumption, better generality, and lower costs. Lu et al. [27] reviewed the MRF polishing technology which had been researched between 2016 and 2020, focusing on its major advantages including recycled MRF, updated abrasives in real time, less tool wear, a stable removal function, and the controlled hardness of the MRF micro-grinding head. In addition, the application methods, operation modes, and structural configurations of the MRF machining equipment were discussed, considering different polishing approaches. Two significant factors to achieve accurate polishing or machining, both the type of platform, such as an MR damper, and the polishing principles, such as a slurry polishing technique, were also discussed. The merits and demerits of various polishing techniques were analyzed through the use of several different approaches: dual rotation MR polishing, magnetic compound slurry, MR solid core rotating polishing, belt MR finishing, multiple polishing heads, MR gear profile finishing, reciprocal MR polishing, and wheel-based MR finishing. Eshgarf et al. [28] analyzed appropriate applications of MRFs corresponding to the field-dependent yield shear stress in terms of the field magnitude and particle volume fraction, by adopting several types of vehicle MR dampers which are operated with the flow mode, shear mode and squeeze mode. In particular, the power to operate MRF application systems was discussed by using three types of batteries, including chemical batteries, physical batteries, and biological batteries. It was demonstrated that the fuel battery included in the chemical batteries shows several advantages: (i) the process of direct conversion of chemical potential energy into electrical energy prevents thermal blockage, (ii) it is easy to deal with and very safe due to the lack of moving components, (iii) it is environmentally friendly due to the production of hydrogen. Liu et al. [29] surveyed and systematically reviewed the progress of medical applications of MRFs, mainly emphasizing six categories: lower limb prosthesis, exoskeletons, orthosis, rehabilitation devices, haptic masters, and tactile displays. With MRFs, one can devise stable limb motions in lower limb prostheses, exoskeletons, and orthoses, flexible muscle trainings in rehabilitation devices, and high transparency and high resolution haptic feedback. In exoskeleton applications, exoskeletons for force/strength enhancement and exoskeletons for motion assistance are included, while for the orthosis applications the knee assistance device and ankle foot orthoses were discussed. Among the applications in the medical field for MRF, the research work on the haptic master was mostly undertaken considering robot surgery, in which a surgeon operating the haptic master can feel the same force or torque occurring in the surgical areas by controlling the magnetic field. In addition, in the robot surgery, a tactile display device using MRF can be used to mimic human tissues or organs by controlling the input current. Aziz et al. [30] presented a review of large-sized MR dampers, which can produce a high enough damping force with a relatively small power to be applied in several industries. The fields of application are classified as civil infrastructure, automotives, gun recoil systems, vibration isolation systems, railway vehicles, powertrain mounts, the polishing industry, and military vehicles. For example, a 400 kN large MR damper was developed for the vibration control of civil structures, a 600 kN MR damper was made based on the hyperbolic tangent function model and applied to the vibration control of building structures with proper control logics. The largest MR damper, which has a capacity of 6000 kN, was made in China and applied to the vibration control of the Binzhou Yellow River Bridge. Most large-sized MR dampers were designed using the Bingham model-based monotube large MR damper (single-ended), the hyperbolic tangent function-based mono tube single-ended large MR damper, the modified Bouc–Wen model-based monotube large MR damper (double-ended), the phenomenological Bouc–Wen model-based monotube large MR damper (double-ended), the phenomenological Dahl friction model-based mono-tube large MR damper (double-ended), the Bingham model-based monotube large MR damper (double-ended), and the Herschel–Bulkley model-based monotube large MR damper (double-ended). 

Do and Choi [31] reviewed six different smart material actuators applied to diverse control applications, considering the aspect of control performances. Among actuators, an MRF actuator was investigated using various application systems including a vibration control automotive suspension system, a vibration isolator, and the tiny vibration control of a precision table. To achieve enhanced performances of these application systems, a proper control strategy, corresponding to the motion characteristics of each application, should be designed. In the review article, several control strategies, which are popularly used for MRF application systems, were discussed, and the following were chosen: a sliding mode controller, a H-infinity controller, a fuzzy controller, a neural networks model-based controller, a model free fuzzy controller, and a phase-based fuzzy logic control. Some advantages and disadvantages of each control strategy were discussed in terms of control performance, practical implementation, cost-effectiveness, signal processing of the feedback states, and wide applicability to diverse MRF applications. Fardan et al. [32] summarized the research trend of auxetic metamaterials, which have the potential to be developed primarily for use in prosthetic devices, protective devices, robotic applications, and aerospace engineering, by treating MRF as one of the metamaterials, since its inherent characteristics are controllable by the input current. Among many applications, the prosthetic design was fully discussed, showing some features such as the negative Poisson’s ratio effect and other relevant properties. Masa’id et al. [33] surveyed various control strategies applied to smart MR materials in a semi-active manner. It is well known that MR dampers can dissipate the energy only, and hence the semi-active controller needs to be implemented to increase the damping performance due to the magnetic field. One representative semi-active controller is the skyhook controller, proposed firstly by Karnopp et al. [34]. Later on, a sky–ground hook controller was developed for the vibration control of both the body mode and wheel mode of vehicles’ suspension systems. In this review paper, each controller was applied to an appropriate application system. For example, LQR or LQG demonstrate high control performances for automotives, railway trains, aero-foil shapes, and building structures, while the sliding mode controller demonstrates good performance when used for several uncertain systems, and the fuzzy logic controller is well adapted to use in military vehicles. Each control system was discussed regarding its advantages and disadvantages, followed by issues to be solved by future works, such as how to make a simple and cheap microprocessor which can be easily implemented in a real-world environment, with relatively small power to operate MR applications. 

Table 2 summarizes the major review articles published in the last two decades. It can be clearly seen that various application devices and systems utilizing MRFs have been proposed and studied, including linear and rotary MR dampers, MR mounts, medical and rehabilitation devices, polishing technologies, and civil structures such as bridge cables. In addition, diverse semi-active control strategies such as skyhook controllers, sliding mode controllers, optimal controllers and adaptive fuzzy controllers are applied to the MRF systems to achieve desirable performances. 

## 4. Temperature Effect and Thermal Conductivity 

As mentioned earlier, one of the most significant issues for successful developments in MRF technology for its use in real-world environments is reducing or eliminating the effect of temperature on the MRFs and the thermal effect in the application systems, to maintain advanced properties and performances regardless of temperature variation. Most MRFs consist of magnetic particles, carrier liquids, and additives, and hence the main characteristic of the MRF itself is its high sensitivity to the temperature induced from the magnetic coil (or input current). Therefore, the heat needs to be effectively dissipated to achieve the maximum performance of the application system, which associates the work of the heat transfer analysis of the design structure with the magnetic circuit position. Furthermore, the temperature issue is closely related to energy consumption during dynamic motions, the maximum input magnetic field due to saturation, and the operational durability. In the subsequent two sections, major research studies on the temperature and thermal conductivity of MRFs and the thermal effect in the application systems are surveyed and summarized. 

Zschunke et al. [35] presented an approach to the solution of the variation reduction due to the magnetic field strength, geometry, and temperature. They used an Arrhenius relationship, in which the fluid viscosity is a function of the shear rate, magnetic field, and temperature. It was shown that the viscosity of the MR fluid followed the Arrhenius expression, stating that the temperature effect depended only on the change in viscosity of the matrix fluid. Heine et al. [36] analyzed the thermal energy transport in sheared MRFs by ignoring viscous dissipation. A particle-level simulation was carried out to determine the suspension structure, in terms of the Mason number and volume fractions. It was demonstrated that a small Mason number resulted in a larger and more effective thermal conductivity than a large Mason number. It was also shown that the effective thermal conductivity of the sheared MRFs could be roughly approximated as double, as the Mason number decreased from the large to the small limit. Ocalan [37] conducted a literature survey in their thesis, focusing on the effect of temperature on the rheological properties of MRFs and the theoretical predictions of changes in the field-dependent properties of MRFs as the temperature varies. The dependence of the magnetic force on temperature arose from the effects of thermal fluctuations on ferromagnetic ordering within the particles, while the ferromagnetic ordering was completely lost at the Curie temperature in the absence of the magnetic field. In addition, the saturation magnetization of the ferromagnetic material was a monotonically decreasing function of temperature at below-Curie temperatures. On the other hand, the Brownian force resulting from a temperature rise from 25 °C to 70 °C only corresponded to a change of half an order of magnitude. The effect of temperature on magnetorheological response was evaluated under three levels of magnetic flux density. The measurements on five samples were taken at three temperatures each: 20, 75, and 130 °C. From this test, it was demonstrated that, at higher flux densities, the current requirement was higher at elevated temperatures, and the high temperature results showed a systematic and statistically significant decline in magnetorheological stress. Yildirim and Genc [38] experimentally studied the thermal conductivity of MRFs synthesized with iron powder and silicone oil, with several particle volume fractions (5, 20, and 40 vol%) of two different grades of iron (Fe) and magnetic field strengths. The thermal conductivity behavior of the MR fluids in different temperature ranges was analyzed and it was found that the heat transfer was more effective at higher temperatures, while the thermal conductivity increased in the temperature range from 0 °C to 50 °C, and from 50 °C to 100 °C, but decreased from 20 °C to 100 °C. This decrease could have been due to the decrease in the thermal conductivity of the silicone oil. As a result, silicone oil-based MR fluids may not be suitable for low temperature heat transfer applications. It was identified that the heat transfer coefficient was high for higher particle concentrations and the percentage increase was more pronounced for lower particle concentrations. This may be due to the close packing of particles in the higher volume fractions of the magnetic phase. For lower concentrations, the particles which are farther away from each other in the off-state lined up in a chain-like formation, and the interaction is increased under the magnetic field. Chang et al. [39] investigated the thermal conductivity of a set of MRFs made of CIPs and three different oils. In particular, the concentration of MRFs was chosen as a key parameter to formulate the relationship considering thermal conductivity. It was shown that there was a linear correlation between thermal conductivity and the concentration of MRFs. Applying this trend, the concentration variation of MRFs can be acquired on the basis of thermal conductivity variation. 

Bilyk et al. [40] formulated a mathematical model of MRF flow in an annular channel of the MR shock absorber, taking into account the forces of dry friction and gas friction in a pneumatic camera, to investigate the dependence of the MRF’s rheological properties on shear rate, temperature, and magnetic flux density. It was observed that the resistance force, with an increase in magnetic flux density, increased forty times, but the resistance force with a growth in temperature from 20 °C to 80 °C decreased seven times in a magnetic field of 500 mT, and twice without the field. This temperature dependence can be used for the development of a control algorithm for shock absorber performance characteristics, which would need to be integrated with the electronic control unit’s practical implementation. Wang et al. [41] investigated the effects of temperature on the material properties of MRF components. Both theoretical analysis and experimental investigation were performed on the temperature-dependent material properties of MRF components. The experiment showed that both the mass magnetization and coercivity of MR particles decreased as the temperature increased, and that this phenomenon was particularly obvious at high temperatures. It was also identified that the shear viscosity and a relatively large thermal expansion of the carrier fluid were severely decreased by increasing the temperature further; also, the magnetization performance of the MR particles declined sharply after a long-term exposure to a high-temperature environment, due to the formation of a surface oxidation layer. In addition, the shear viscosity of the carrier fluid decreased as the temperature increased, and a carrier fluid with a higher viscosity was more sensitive to the temperature variation. Mistik et al. [42] investigated the compression effect caused by the temperature of MRF contained in a fabric subjected to a magnetic field. The compression test was carried out on MRF-filled spacer fabric, with and without a magnetic field in a temperature range from 15 °C to 70 °C. The thermal conductivity of the MRF-filled spacer fabric was also tested to see the effect of the change in the magnetization and rigidity. The compression tests exhibited an increase with an increase in the displacement value, showing the intensity of the temperature dependence. On the other hand, it was seen that the encapsulation of the MRF had a considerable effect on the thermal conductivity of the spacer fabric, resulting in a nearly fivefold increase in the thermal conductivity of the spacer fabric, even in the absence of an applied magnetic field. Chen et al. [43] presented the effect of temperature on the shearing transmission performance of an MR transmission device under a external magnetic field, and also investigated the influence of temperature on the shearing stress and transmission performance. It was demonstrated that the torque transferred by the MRF gradually declined as the temperature rose under the same magnetic field strength and rotating speed, but the variation was continuous and uniform. Once the temperature was higher than 100 °C, the torque transferred by the MRF gradually increased and varied irregularly, which caused an unstable transmission performance, due to the thickening behavior of the MRF after the temperature rose above 100 °C. 

Rahim and Ismail [44] investigated the thermal behavior of MRF and nanofluid, focusing on the understanding of their thermal conductivity properties via several parameters, including particle volume fraction, the shape and size of particles, the material of the particles, the base fluid, and a magnetic field. It was shown that an increase in particle volume fraction and magnetic field strength may have increased the thermal conductivity of the MRF. From the theory and experiment given, the relationship between these parameters and thermal conductivity can be found. Although there are several studies on the enhancement of thermal conductivity of MRFs from 0.42 W/m K to 0.54 W/m K, there is still room for improvement to enable the optimization of the fluids to be used in practical applications. The potential applications of magnetorheological fluids with better thermal properties would be thermomagnetic convection and heat pipes. Sherman et al. [45,46] studied the temperature-dependent behavior of the carrier fluid, applying non-Brownian suspension theory. To carry this out, two fluids were sheared with the same carrier fluid, then their fluid properties were scaled in temperature similarly. This method was first validated by measuring the viscosity across temperature for custom model fluids (commercial MRFs), to conform to the theory showing the temperature scaling was within 5% for both the MRFs and their carrier fluid. It was shown that the MRFs exhibited common scaling to within 4% and that the effect of magnetic hysteresis was negligible in yield stress increment. The proposed measurement method of the viscosity across temperature for a pair of custom MRFs and their corresponding carrier liquid was very useful to identify the temperature dependence of MRFs in fewer experiments. Yang et al. [47] carried out experimental research in the investigation of the hyperthermia effect of magnetic functional fluids using three samples: Fe_73.5_Nb_3_Cu_1_Si_13.5_B_9_ amorphous particles, CoFe_2_O_4_ nanoparticles, and Fe_3_O_4_ nanoparticles dispersed in water. It was observed from the hyperthermia experiment that, when the alternating electrical current was 150 A, the temperature of the functional fluids based on amorphous particles could rise to 33 °C in 1500 s. When the current was 300 A, the final stable temperature could reach 20 °C to 80 °C (60 °C). This study demonstrated that the Fe_73.5_Nb_3_Cu_1_Si_13.5_B_9_ magnetic functional fluids may have potential for biomedical applications. The hyperthermia experimental results indicated that the Fe_73.5_Nb_3_Cu_1_Si_13.5_B_9_ MRF exhibited a more significant heating effect than that of CoFe_2_O_4_FF and Fe_3_O_4_FF in an alternating magnetic field. As a result, it was shown from the hyperthermia experiment that the Fe_73.5_Nb_3_Cu_1_Si_13.5_B_9_ MRF had the best potential for the hyperthermia therapy of tumors in biomedical applications. Rabbani et al. [48] studied the stability and rheological properties of MRF that consisted of carbonyl iron microparticles and silicone oil, within a temperature range of 10 °C to 85 °C. During the preparation of the MRF, 3 wt% of stearic acid was added to enhance fluid stability, and resulted in 92% stability enhancement for a period of a month, which was eight times more than common MRF. It was also shown that as the temperature increased, the viscosity and maximum yield stress decreased. The investigation of the type and weight fraction of MRF additives and also particle polydispersity on the MR effect and MRF stability at high temperatures over 80 °C could be the significant subject of future research. Shah et al. [49] presented the field-dependent rheological properties of a nano-sized magnetic particle-based ferrofluid (NMPFF), investigated in dynamic mode at different temperatures. The NMPFF had several inherent rheological properties such as improved shear thinning, elevated dynamic moduli, and thermo-rheological complexity. These properties came from the presence of a long chain-like structure in the NMPFF, under the influence of an applied magnetic field. The proposed NMPFF was a magnetic colloidal suspension of a mixture of two different nano-sized magnetic particles, which were dispersed in a carrier liquid in an appropriate weight fraction. Both the structural and morphological properties of the particles were firstly investigated using small-angle neutron scattering, and a transmission electron microscope was observed. Subsequently, the temperature-dependent rheological properties of the NMPFF were measured in an oscillatory mode using the magneto-rheometer. It was shown that the crossover strain value increases with the augmenting temperature at the low field, and it decreases with the increasing magnetic field strength. It was also found from the frequency sweep test that a slight change in the magnitude of the storage modulus with the increased temperature and magnetic field strength occurred. 

Rahim et al. [46] investigated the thermal issue, which needs to be seriously resolved in the heat dissipation technology, such as MRF filled inside a PMMA (polymethylmethacrylate), by carrying out the measurement of the thermal conductivity in both the parallel and perpendicular orientations with the magnetic field. The magnetized distribution was undertaken using the finite element method (FEM), and three parameters were looked into, namely, the PMMA diameter, PMMA height, and gap thickness. By utilizing the FEM software, the simulation results were produced, and magnetic flux density for all of the designs was achieved. It was clearly found that the gap thickness played a significant role in determining the optimal design. For both vertical and horizontal magnet arrangements, the highest magnetic flux densities were produced with the gap thickness at 5 mm. Forero-Sandoval et al. [50] studied the thermal conductivity and viscosity of MRF composed of CIP immersed in silicone oil. A thermal-wave resonant cavity was employed to measure the thermal diffusivity of the MRF as a function of an externally applied magnetic field. The dynamic viscosity was also measured, and its relationship with the concentration of the particles and the magnetic field strength was investigated. It was shown that higher concentrations of CIPs as well as higher magnetic field intensities lead to a significant increase in the thermal conductivity. Thus, the relationship between the thermal conductivity and the dynamic viscosity can be explored using highly viscous materials. It has been also found that the addition of CIP combined with the magnetic field induce the formation of chains that significantly increases the thermal conductivity and viscosity. Rahim et al. [51] found that the geometries of the PMMA container indicates a minimal flux density change at all parameter levels. For the vertical magnet arrangement, the low level of the PMMA diameter resulted in a higher flux density regardless of the PMMA height, and this is a complete opposite to the horizontal magnet arrangement in which the PMMA height at a low level (30 mm) was more dominant, irrespective of the PMMA diameter. Therefore, it was concluded that the optimal design for the thermal conductivity measurement instrument was possible by keeping the PMMA geometries at a medium level with a 5 mm gap thickness. McKee et al. [52] studied the temperature effect on the performance of compressible MRF. MRF is a temperature-dependent material, where its compressibility and rheological properties change with the temperature. Firstly, it was found that the shear yield stress of the MRF remains unchanged within the testing range while both the plastic viscosity, using the Bingham plastic model, and the bulk modulus of the MRF decrease as the temperature of the fluid increases (25 °C~70 °C). A theoretical model integrated with the temperature-dependent properties of MRF has also been proposed to predict the behavior of a compressible MRF. It was demonstrated in the experimental results that the proposed model has good accuracy to predict the stiffness and energy dissipation of the compressible MRF, showing that both the stiffness and the energy dissipation decrease with an increase in the temperature of the MRF. Maroofi and Hashemabadi [53] adopted the Discrete Phase Model (DPM) to simulate the effects of the influencing parameters on the thermal conductivity of MRFs, and the computer fluid dynamics (CFD) simulation results are validated with the experimental data from Transient Hot Wire (THW). It has been shown that the magnetic field intensity variations are a proper factor for changing the thermal conductivity of the MRF, and if the external magnetic field is parallel to the temperature gradient, the chains of the magnetic particles act as facilitators of heat conduction, increasing the thermal conductivity of the MRF. It has been identified that the conditions of larger dispersed particles, the volume fraction, an intensified applied magnetic field, and smaller dispersed particles increase the thermal conductivity anisotropy in the MRFs. Therefore, the thermal conductivity of MRFs is a controllable property that can be increased or decreased based on the direction of the magnetic field, magnetic field intensity, and size and volume fraction of the dispersed particles, and this salient characteristic can control the thermal effect and heat transfer in MRF application systems. 

Maroofi et al. [54] studied a variation of thermal conductivity of the MRF via the visualization of the cluster formation inside the fluid, which can be achieved via CFD simulations. It was shown that when more and longer chains of particles are formed, the variation in the thermal conductivity of the MRF is more considerable. In addition, it was identified from an experimental test that the thermal conductivity of the MRF with a 15% dispersed particle volume fraction is increased to 117% by applying a 11.16 kA/m magnetic field. Thus, it was found that whenever the magnetic field strength and the dispersed volume fraction are greater and the size of the particles is smaller, the thermal conductivity of the MRF is further increased. Pisuwala [55] presented the thermal properties of MRFs made of transformer oil and 2 wt.% of Fe_3_O_4_ particles. Firstly, it was shown that the thermal conductivity is enhanced by 219%, 304%, and 356% by changing the iron concentration from 15 vol.%, 20 vol.%, and 25 vol.%, respectively. This increment is higher than the normal spherical-shaped iron-particle-based MRFs. The increase in the thermal conductivity of the transformer oil-based MRF is due to the shape effect as well as the reduction in the thermal resistance due to a higher particle–particle interaction compared to spherical particles. In addition, when adding nanoparticles, the friction between the particles is reduced, and the nanoparticles facilitate flake-shaped particles to form a stronger chain. This results in an enhancement in the thermal conductivity. Therefore, the observed reduction in the viscosity of MRFs with nanoparticles supports the concept of the increased orientation of flakes in the direction of the field. It was concluded that the observed enhancement in the field is due to the shape of the particles, the increase in the ordering of particle chains, and the reductions in the friction and viscosity of the MRF with the nanoparticles. Hemmatian et al. [56] investigated the influence of temperature on the linear and nonlinear viscoelastic behaviors of MRFs. In this work, the shear flow and oscillatory shear strain experiments were conducted over a wide temperature range (−5 to 50 °C) for different levels of magnetic flux density, strain amplitude, and rate. Firstly, it was shown that the MRF is highly dependent on the temperature, and the shear stress increases when the temperature is reduced, especially below 10 °C. Moreover, it has been observed that applying the magnetic field decreases the effect of the carrier fluid, and consequently, the dependency of the MRF on the temperature decreases. In addition, it has been shown that in the linear region, the temperature mostly affects the storage and loss moduli in the absence of the applied flux density. On the other hand, the influence of the driving frequency on the viscoelastic properties of the MRFs was studied, resulting in the increment in both the storage and loss moduli as the frequency increased in the absence of a magnetic field. Li et al. [57] studied the influence of a temperature increase on the properties of MRFs using different constitutive models, considering the temperature rising from 20 °C to 70 °C. In the viscosity test, the zero-field viscosity of the MRFs decreased with the increasing temperature, as expected, and this phenomenon was more obvious at higher shear rates. The shear stress of the MRFs also decreased significantly with an increasing temperature in the presence of the magnetic field, and a further decrease appeared at higher shear rates. It has been shown that the Herschel–Bulkley model, which has a high accuracy at room temperature, is no longer able to accurately describe the shear stress of MRFs at high temperatures, causing an error up to 21.4% at 70 °C. The decrease in the shear stress caused by the temperature rises is mainly due to the change in the hydrodynamic force between the particles and base carrier fluid. The hydrodynamic force follows the Navier–Stokes law, where the temperature-dependent viscosity of the base carrier fluid follows the Andrade formula. Therefore, a new constitutive model with temperature prediction was formulated and tested, showing a small error at different temperatures and magnetic field strengths. Xiong et al. [58] investigated the temperature effect by making a yield stress testing device for MRFs, in which the temperature field model is considered including the enameled wire and assembly gap. And simulations were carried out to compare the results with the measured results of the thermal conductivity of the coil winding and assembly gap. Then, the following results were drawn: (i) When the temperature field reaches approximately 90 min, the steady-state balance is reached. (ii) The greater the thermal conductivity of the winding, the stronger the thermal conductivity of the surrounding material, and the lower the maximum temperature of the testing device. As the gap increases, the heat dissipation of the testing device decreases, the steady-state temperature rises, and the average gap increases by 0.01 mm. (iii) The thermal conductivity of the enameled wire is determined to be 3.06 W/m^2^K, and the assembly gap is 0.03 mm. Kariganaur et al. [59] presented the temperature effect of the MRF on performance, while the damper device subjected to different load parameters was working using an embedded thermocouple. The results reveal that the fluid temperature rises significantly to 125.39 °C from the atmospheric temperature, with a decrease in the damping force of 66.32% at higher loading parameters. More specifically, the following results were obtained: (i) From the sedimentation measurements, the sedimentation rate was found to be reduced exponentially with the rise in the temperature of the MRF. (ii) The viscosity of the MRF decreases exponentially as the temperature increases, and it increases significantly with an external applied magnetic field strength. (iii) The reduction in the damping force as the temperature increases is an exponential drop, with R^2^ = 0.9989. The saturation point of magnetization before the synthesis of MRFs reveals an 8.28% decrease at higher temperatures compared to the atmospheric temperature. (iv) The heat treatment study of CIP showed that the destabilization of the damper is possible if the system operates at higher temperatures for a prolonged time.

Ji et al. [60] studied the sedimentation stability, expansion rate, volatilization rate, and temperature–viscosity property of MRFs in a high-temperature environment. The MRFs were composed of soft magnetic particles, surfactants, and the base carrier liquid, since both the soft magnetic particles and base liquid influence the properties of the MRF at high temperatures. It has been shown that the proposed MRF has excellent sedimentation stability, of which the sedimentation rate is only 4.42% after a heat treatment at 150 °C for a one-week placement. It has also been observed that at 30 °C, the shear yield stress of the novel fluid is 9.47 kPa under a magnetic field of 817 mT, which is higher than the general MRFs with the same mass fraction. The yield shear stress is less affected by the high-temperature environment, reducing by only 4.03% from 10 °C to 70 °C. Zakinyan et al. [61] investigated the effect of thermal percolation in a magnetic fluid composite by changing the contents of conducting microparticles in a low-conducting medium. It has been shown that the effect of the magnetic field on the thermal conductivity of the composite material is significant at the concentration of conducting microparticles above the percolation threshold and is much less pronounced otherwise. The relative increase in the thermal conductivity can exceed 600% under the influence of a magnetic field parallel to the heat flux and it is achieved in fields of intensity. In addition, it has been found that the magnetic field parallel to the heat flux in the sample significantly affects the thermal conductivity of magnetic fluid composites. On the home page of Lord Company [62], several types of commercial products of MR fluids such as MRF-122EG, MRF-132DG, and MRF-140CG are provided. On this home page, two questions on the temperature problem of MRFs appear: (i) How does temperature affect MRFs? (ii) Is the heat dissipation an issue if MRF applications are used for a long-term operation? For the first question, the answer was written as follows: In the off-state (no magnetic field applied), the temperature effects are largely dependent on the liquid carrier fluid. Oil and silicone oil-based fluids can typically operate from −40 to 150 °C. Water-based fluids can operate from 0 to 70 °C. Glycol can extend water-based fluids to operate below normal freezing temperatures. In the on-state (magnetic field applied), the magnetic viscosity effect is typically an order of magnitude greater than the temperature viscosity effect, and so the device’s performance is uniform and controllable across a wide range of temperatures. However, there is no information on the specific properties of MRFs at lower temperatures ranging from 0 °C to approximately −40 °C. As for the second question, the following answer was given: The heat dissipation depends on whether the damper (device) is also being stroked. If the damper coil is energized to produce the magnetic field, but the piston is not moving, no energy is dissipated, and there is no substantial generation of heat. When the damper is on and the piston is moving, the energy is dissipated as heat. When stroking the damper on a test machine under a constant current for extended periods of time, active cooling may be required to keep the damper from overheating. For example, the RD-1005-3 MR linear damper reaches 120–140 °C (external body temperature) if it is continuously stroked at 2 Hz and +/− 0.5 inches at a 0.5 amp current in a room-temperature environment with no active cooling. The recommended upper temperature for continuous use is 160 °F (70 °C). The heat dissipation heavily depends on the mechanism configuration of applications and the number of magnetic coils (required power). In addition, an exact analysis of the temperature effect on the dynamic or control performances of MRF applications depends on the location and number of temperature sensors such as a thermocouple.

Table 3 summarizes the principal results of major studies on the temperature effect on MRFs, describing the problems and methods to reduce the drawbacks due to the temperature effect. In the middle of the 2000s, both the Arrhenius relationship and Mason number were used to identify the thermal characteristics of MRFs and the relationship between the thermal conductivity and Mason number, and the saturation magnetization and Brownian suspension theory. After that, several methods to release the temperature problem have been proposed by several scholars: the addition of nanoparticles, the addition of nano-sized additives, the use of non-spherical particles, the use of new constitutive models incorporating the temperature effect, and the proper choice of carrier liquids, which are typically operated in a range, from −40 to 150 °C. It is noted here that despite the many methods to reduce the temperature effect, there exist trade-off properties such as the reduction in the viscosity and degradation of MRF application systems, which will be discussed in a subsequent section.

## 5. Thermal Effect in Application Systems

Among numerous applications, as summarized in Section 3, MR dampers are mostly studied and commercially available for automotive suspension systems. So far, various types of MR dampers in terms of the structural configuration and magnetic circuit core have been applied for the vibration control of the vehicle suspension system and the vibration control of civil structures such as bridge cables and many types of prostheses, including legs. However, one of the significant drawbacks of MRF application systems including the MR damper is the weakness of the operation in a wide range of temperatures, which results in the performance degradation of the applications. Breese and Gordaninejad [63,64] firstly studied the heat generation and dissipation of a field-controllable MR damper via both experimental and theoretical studies. It is well known that MR dampers are energy-dissipating devices, and hence, the issues of heat generation and dissipation are important. As a first step, by applying various types of sinusoidal input motions to the damper, both the heat dissipation and heat generation within the MR damper were tested and compared with the proposed theoretical model developed to predict the temperature increase in the MR dampers. It was demonstrated that the theoretical model well predicts the measured results, showing good accuracy, and a non-dimensional form of the governing equations WAS developed to assess the effect of physical parameters on heat dissipation and heat generation. Batterbee and Sims [65] presented the effects of temperature variation on the MR damper in the temperature range from 15 °C to 75 °C by identifying the meaningful parameters of MR damper model while considering the temperature. It was firstly shown that the analysis from the model provides a 34% drop in the viscosity, a 22% reduction in the yield stress, and a 300% increase in the damper stiffness, with a rising temperature under certain conditions. Secondly, it was shown that the temperature effect on the broadband excited single-degree-of-freedom mass isolator is evaluated by adopting a proportional–integral–derivative (PID) controller and gain scheduling and an on/off controller. From this test, it was identified that RMS acceleration is enhanced, but the RMS working space is degraded by increasing the temperature for each controller. Wilsom and Wereley [66] investigated the variation of the pressures of the fluid and the pneumatic accumulator due to the operating temperature. In this work, a theoretical model, which can capture this change in stiffness due to self-heating while preserving the connection to the physical realization of the MR damper, was proposed and validated while considering the temperature range from 0 °C to 100 °C. It was observed that the controllable yield force was found to decrease by up to 20%, the post-yield damping was found to decrease by over 60%, and the stiffness at high piston velocity was found to increase significantly. In addition, the proposed model considering the temperature effect was well validated to capture better stiffness behavior over a large operating temperature range. Zhang et al. [67] presented the multi-physics of a new MR damper with a multistage piston and independent input currents. In order to analyze the multi-physics problem, the COMSOL software was used, considering the electromagnetic, thermal dynamic, and fluid mechanic properties. To evaluate the performance, the measurement index involving the total damping force, dynamic range, and induction time needed for magnetic coil was put forward. For a thermal analysis, it is considered that the heat is transferred in both the solid and the fluid domains and generated from the self-inductance of the electromagnetic coil with the fast-changing current and friction of the relative motion between the piston and the cylinder. It has been found that the majority of the temperature rise is caused by the friction inside the MR damper rather than the self-induction from electromagnetic coils, and the maximum temperature occurs in the piston head around the coils. In addition, it has been identified that the temperature appears to be exponentially rising in the working gap at the beginning, and it slows down when the maximum temperature reaches about 45 °C due to the low thermal conductivity of the MRF. 

Saravanakumar and Ravikumar [68] studied the dynamic characteristics of MR squeeze film dampers used for the rotating balance (or vibration) of an aircraft jet engine rotor. As a first step, tests were conducted for both the viscosity and dynamic characteristics due to the temperature increment, showing that both characteristics were found to decrease with the increase in temperature. Then, the temperature effects and the length/diameter (L/D) ratio effect on the MR squeeze film dampers were tested while considering the zone of high temperature gradients and high heat generation due to the current in the magnetic coils. It was found that the dynamic characteristics decrease with the increase in the shear strain rate but improve when the magnetic field intensity is increased. This behavior tends to deteriorate when the temperature is increased as the viscosity decreases. Thus, it has been suggested that both the damping and stiffness coefficients of the squeeze film damper can be enhanced by increasing the magnetic field intensity. Ferdaus et al. [69] studied the temperature effect on the performances of MR dampers since conventional MR dampers normally face wide variations in temperature due to the magnetic coils attached in the piston head, resulting in the creation of heat. It has been shown that the fluid viscosity has a direct relation with temperature, and hence, the dynamic performances of MR dampers. One of the possible methods to resolve the temperature effect is to change the design configuration by positioning the electromagnetic coils to minimize the heat generation under the magnetic field. As for the MRF itself, the use of a carrier liquid that is not sensitive to the temperature is one of the possible solutions. Yu et al. [70] formulated a theoretical model of the temperature change in a self-decoupling magnetorheological (SDMR) damper, and the prototype was manufactured to test the heat dissipation requirements. As a first step, the temperature performance test was undertaken, showing that as the temperature rises, the damping force declines significantly, since high heat generation occurs due to the operation of the large-sized SDMR damper that is applicable to wind vibration control in civil engineering. In addition, it has been suggested that finding the optimal locations of the magnetic circuits and conducting an exact analysis of the heat transfer of the MR damper depends on the geometrical configuration with proper dimensions. Huang et al. [71] proposed a transmission method of MRF in disc type driven by a shape memory alloy (SMA) spring, with the thermal effect and tis torque responses being due to the variation in the temperature. The flow and transmission of the MRF between two discs were analyzed, and the equations of the flow velocity and transmission torque of the MRF between two discs were obtained. It has been found that the MRFs that flow between two discs can be approximated to laminar flow, and the transmission torque increases with the applied magnetic field. On the other hand, the MRF was driven to the work chamber via the SMA spring, and hence, the electric current was also adjusted by the temperature. Therefore, the transmission torque of the MRF is consecutively controlled, and the value of it increases with the temperature. Sarkar and Hirani [72] investigated the heat transfer when the MRF was operated like a brake friction motion by assuming that the heat generation inevitably occurred during the braking action. In this work, an MRF with nano-silver and nano-copper particles was synthesized and investigated on the characteristics of the heat transfer rate and the demagnetizing effect of those particles on the shear stress of the MRF. Five different MRFs, containing different percentages of silver and copper nanoparticles, were synthesized. The shear stresses of all five MRFs were measured using a magneto-rheometer, and the flywheel-based MR brake experimental setup was used to analyze the performances of the synthesized MRFs. “T” type thermocouples were used to measure the temperature distribution of the fabricated MR brake. The results of the temperature distribution of brakes containing five synthesized MRFs were measured and compared. In addition, the maximum temperatures on the MR brake housing surfaces of five different MR fluids were measured, and it was found that the temperature differences (maximum temperature reading-minimum temperature reading) for MRF85 (without silver particles), MRF85_0.25Ag (0.25% silver particles), MRF85_0.5Ag (0.5% silver particles), MRF85_0.25Cu (0.25% copper), and MRF85_0.50Cu (0.5% copper) were identified by 18 °C, 12 °C, 14 °C, 16 °C, and 9 °C, respectively. Therefore, the increase in the silver nanoparticle percentage helps to achieve a uniform temperature distribution on the MR brake. The difference in the temperature around the periphery of the disk of the MR brake may be due to misalignment between the disk and the MR brake housing. It was also observed that the difference in the MR gap and non-uniform distribution of MR particles caused the non-uniform distribution in temperature. 

Paul et al. [73] studied the vibration reduction when the MRF was used for metal cutting. In metal cutting, the MRF is used to reduce the tool vibration, but a major problem in this case is that both durability and temperature have effects on the MRF properties. This work proposed one method to reduce the temperature and to improve the viscosity of MR fluids by infusing nanoparticles along with conventional MRFs. Both aluminum oxide and titanium oxide nanoparticles of 0.1% and 0.2% concentration by weight were considered, and experimental tests were conducted to investigate the influence of nanoparticles on the performances of MRFs. In the cutting process, major parameters such as the cutting temperature, cutting force, surface roughness, and tool vibration during the hard turning of AISI 4340 steel were considered. Two different nanoparticles, Al_2_O_3_ and TiO_2_, with weights of 0.1% and 0.2%, respectively, were used separately, and series of experiments were carried out, with the following results: (i) The impregnation of the nanoparticles can provide better stability, higher viscosity, and better cutting performance during the hard process. (ii) To achieve better damping capability and cutting performance, the damper should be made with MRFs impregnated with titanium oxide ferro and magnetized with a direct current of a higher magnitude. (iii) Nanoparticles used along with MRFs reduce the cutting temperature in the MRF, especially outside the core. Mitrouchev et al. [74] proposed a methodology to calculate some principal characteristics of the damper such as the electromagnet’s magnetic field value, emitted heat, and damping force. The methodology was based on analytic calculations of the characteristics and a finite element method analysis. It was found that two main geometrical characteristics of the damper, namely, the piston thickness and electromagnet width, were optimally chosen, thus allowing for the maximum damping force to be reached. In addition, it was identified that the heating of solenoid is the biggest uncertainty at the highest voltage. Specifically, the maximum power of the electromagnet was identified as 15 W when the heat did not exceed 100 °C within 30 min of continuous operation, where the maximum damping force was reached at a piston thickness of 10mm and an electromagnet width of 26 mm.

Parlak et al. [75] experimentally investigated the performances of MR dampers while considering specified parameters including the gap width, the active length, the gap length, and the radius of the piston core. The tests of the nine manufactured dampers were carried out under a constant damping velocity at 300 °C, and they were checked with the thermocouples placed in the piston head, where the varying temperatures’ effects on the performance of the damper are the most significant, resulting in the degradation of the damper responses. It was shown that the effect of the gap width, which is the most dominant, increases with the increasing velocity by just 5.56%, while the effect of the active length decreases by 5.13% to achieve the maximum damping force. In addition, it has been found that the effects of all parameters are almost unchanged with the velocity for the maximum dynamic range, directly indicating that both the velocity needed to achieve the maximum force and the dynamic range could not be realized in a real environment. Wang et al. [76] investigated the effect of temperature on the transmission characteristics of high-torque MR brakes by theoretically analyzing a disc brake, the pressure characteristics of the MRF in an axial direction, and the heat dissipation. A high-torque squeezing MR brake equipped with a water cooling system for heat dissipation was designed and manufactured. It was found that even though the MR brake was manufactured to be operated in desired workable pressure characteristics for a high-torque transmission range, the braking torque exhibited a variation trend that initially increased and then decreased when the temperature increased from 25 °C to 150 °C. The magnitude of decrease was approximately 210 N m, as it decreased from 1800 to 1590 N m. It has been also observed that the temperature of the MRF presents an almost linear increase at different slip powers, and the rate of the temperature rise increases with an increase in the slip power. The results presented in this work directly indicate that an effective cooling method can be vital to the stable operation of the heat dissipation in high-torque and high-power MR devices including a high-torque MR brake. Gołdasz and Sapinski [77] carried out a numerical study to analyze both heat generation and heat dissipation mechanisms within an MR damper operating in squeeze mode. In this numerical experiment, an exemplary squeeze-mode device was evaluated for temperature changes (0 °C~40 °C) resulting from mechanical and electrical inputs, respectively. The proposed model based on the Stefan model was coupled while considering the lumped parameters of the electromagnetic circuits. It was shown that the obtained data reveal the effects of the temperature rise and force changes upon stroking, and hence, control studies involving operating and environmental conditions such as long operation times and low-temperature (cold) warm-up cycles should be carried out in the future. Song et al. [78] reviewed the thermal and tribological properties of a cylindrical MR brake operated using the shear mode. As a first step, a theoretical analysis of the heating and heat dissipation of the MR brake was performed, and the transient temperature behaviors of the MR brake were simulated using the finite element method. It was identified that the increment in the temperature of the MRF domain is heavily dependent on the working gap. The smaller the working gap, the higher the increment in the temperature. This directly indicates that when the working gap is very small, an appropriate cooling measure needs to be adopted to avoid the degradation of the braking performances. In addition, several heating and wear tests were conducted on the MR brake, and worn surfaces of the friction plates were observed using SEM to understand the tribological characteristics of the MR brake. It was shown from the results that the working gap significantly affects the wear property and tribological characteristics, and these behaviors cause the performance degradation of the MR brake. 

Versaci et al. [79] presented a steady-state magnetic induction field with associated steady-state thermal stresses and a consequent constant yield stress of the MRF using the finite element method. Since a steady-state electric current circulates in the coil of the device, a magneto-static analysis of the device, particularly of the strip of the MRF contained therein, is conducted since the device is subject to heating during operation, and a thermo-static analysis is also carried out considering different external temperatures. Since MRFs change their rheological behaviors as the absolute temperature, T, changes, and the MR damper is subject to temperature changes due to climates of intense cold or particular overheating conditions, a thermostatic analysis needs to be carried out to evaluate the temperature distribution. It has been observed that the influence of temperature on both the magnetization of the particles and the MRF is significant in the MRF strip. On the other hand, the coercivity decreased as the temperature increased, which was mainly due to the fact that the temperature rise accelerates the atomic motion and therefore disorders the magnetic moment orientation of the particles, which results in a reduction in the magnetic moment. In addition, it was identified that the influence of temperature affects its global viscosity when the temperature is increased. Liu and Wu [80] analyzed the mechanical properties of the two special dampers installed in the naval gun test shell using the FEA and CFD models. It was found that both models analyze the dynamic characteristics of the MR damper and colloidal damper well. The results of the quasi-static experiment and simulation especially have a high matching degree, which proves that the model is accurate. The energy absorption rate of colloidal dampers under impact is 97.8%, while the MR damper has a higher energy absorption rate, which reaches to almost 100%. In addition, it was shown that multiple vortices with different sizes appeared in the MR damper during the dynamic impact process, while the streamline of the colloidal damper was stable without vortex generation. On the other hand, the heat of the damper was mainly generated in the annular gap. Due to the improved fluidity, the final temperature distribution of the MR damper was more uniform, and the temperature of the colloidal damper was concentrated near the annular gap. This means that the MR damper has higher resistance peaks, a longer recovery time, and a lower recovery velocity compared with the colloidal fluid damper. Du et al. [81] proposed a new MR damper by adding an aluminum foil bubble insulation material with a low thermal conductivity in the cavity between the electromagnetic coil and the MRF to avoid a rapid rise in the temperature of the MRF in the working process of the damper. First, the temperature-dependent rheological properties of the MRF were tested using a rheometer, and squeezing and stretching tests were conducted at different temperatures to identify the relationship between the dynamic viscosity and the shear stress, squeezing stress, stretching stress, and temperature. It was found that the dynamic viscosity decreases when the temperature and shear rate increase. The fitting formula presented in this paper can reflect the change in the dynamic viscosity of the MRF with the temperature and shear rate. In addition, the squeezing stress and stretching stress decrease with the increasing temperature, and the amplitude of decrease will increase with the increasing strain. When the temperature of the electromagnetic coil rises from 30 °C to 70 °C, the numerical simulation shows that, compared with the rate of increase in the MRF of the damper without the heat insulation device, the rate of increase of the damper with the heat insulation device will decrease by 57.4% in the working area. In addition, with the increasing temperature of the electromagnetic coil, the temperature decreases by 22.9~46.0% with insulation, and this difference will continue to increase with the temperature of the electromagnetic coil. It has been also identified that the rate of increase in the MRF temperature in the working area of the damper with the insulation material could be reduced by 57.4% compared to that of the damper without the insulation material. Parlak et al. [82] identified the relationship between the damping performance and temperature in the control algorithms using a new methodology, which considers the dynamic behaviors of the MR damper depending on the temperature based on the Bouc–Wen model. After evaluating seven parameters in the Bouc–Wen model related with the temperature, the damping force was defined depending on the temperature, with a single equation that significantly simplifies the control process. The temperature considered in this work ranges from 20 °C to 70 °C. It was demonstrated that the damping force error evaluated from the experiment and the model is small, varying from %0.89 to %8.4. The reduction in the damping force of the MR damper was caused from both the reduced viscosity of the carrier liquid and the reduced yield stress of MRF as the temperature increased. Chen et al. [83] investigated the heat dissipation of high-power MR transmission devices owing to wall slip, particle friction, and exciting current by utilizing a new water-cooling structure in which a negative effect of centrifugal force on heat dissipation is observed. The magnetic circuit of the MR transmission device was designed on the basis of the electromagnetic theory, and the magnetic field was investigated via a finite element calculation using the exciting coil with a diameter of 1 mm and turns of 2400. It was shown that the magnetic field strength increases with the exciting current and decreases with the increase in the width of the working gap, but it does not change significantly when the magnetic conduction arc becomes thicker. The proposed MR transmission device is set as the designed maximum transmission power (7.5 kW) to verify its heat dissipation effects under extreme conditions. After cooling water flows into the cooling channel, the temperature of the MRF in the device drops sharply from 70 °C to 28 °C, decreasing by 60%. It was found that the power transmission device has an excellent heat dissipation effect, indicating better performance, considering new structures with the radius and of the magnetic conduction arc and the number of transmission discs. 

Xiao et al. [84] investigated the transient temperature distribution pattern inside the magnetorheological MR grease torsional vibration damper. As a first step, a theoretical heat transfer model of the MR torsional vibration damper with a dual heat source structural feature was established based on the Bingham constitutive model and the temperature-dependent viscosity characteristics. Form the theoretical model, it was shown that the temperature increment in the MR grease in the working domain is the fastest, but there is a gradual slowing in the temperature rise rate. The magnitude and rate of temperature rise are at their maximums when the 1 A current is applied to the torsional vibration damper. The current–temperature curve was obtained by fitting the simulation results. The results of the analysis reveal the internal temperature distribution and temperature rise characteristics of the torsional vibration damper. In addition, it was demonstrated that the temperatures of both end surfaces gradually increase from the center of the circle along the radial direction. The maximum temperature of each working condition was collected and fitted to obtain the relationship between the current and temperature. By analyzing the five working conditions, when the applied current is 0~1 A, the increase in the MR grease viscosity leads to an increase in the rate and magnitude of temperature rise inside the device. Jacob et al. [85] presented a hybrid damper concept using a combination of MRF and shape memory alloy (SMA)-based energy dissipation. In this work, it was demonstrated that the operation of MR and SMA dampers complement each other to compensate for each other’s weaknesses. In particular, the slow response from the MR damper is compensated by passive SMA damping using the pseudoplastic effect of martensite reorientation, which can dissipate the significant amount of shock energy at the beginning of the shock occurrence. The inability of the SMA to continuously dampen the subsequent vibration was taken care of by the MR damper, which could dissipate the energy as long as a magnetic field was applied. Therefore, the SMA damper can be used in wider applications, while the energy consumption of the MR damper can be reduced. The energy needed for the MR damper decreases with increasing shock loading amplitudes, showing improved settling behavior owing to the enhanced energy dissipation by the SMA damper. Specifically, a reduction of up to 96% in the energy consumption was achieved for the MR damper, as the current excitation requirement was reduced from 5 A to 1 A. This mutual integration concept in a hybrid form can open the possibility of reducing the MR damper’s construction volume and the temperature effect. Szelag et al. [86] proposed a field model of coupled phenomena occurring in an axisymmetric MR brake focusing on transient fluid dynamics, electromagnetic properties, and thermal fields. The influence of temperature on the electromagnetic and rheological properties of the used materials has also been taken into account using the finite element method and a step-by-step algorithm to figure out the coupled phenomena considered for the MR brake. The nonlinearity of the magnetic circuit and rheological properties of the MRF as well as the influence of temperature on the properties of the materials have been tested, and the Newton–Raphson method and the coupled block over-relaxation method were implemented. The elaborated algorithm has been successfully used in the analysis of the phenomena in the considered MR brake. The accuracy of the developed model has been verified, and its usefulness has been demonstrated via a comparative analysis of the results of the simulation and laboratory tests of the MR brake. It was also remarked that the successful development of this technology required the durability of the MRF in a wide temperature range and the robustness of the applied systems. 

Table 4 presents a summary of the temperature effect on the working performances of various MRF application systems. It is clearly seen that MRFs are applied to special systems such as a high-power transmission system to investigate the heat generation and temperature distribution. Many studies have focused on the several methods used to reduce the thermal effect during the working operation. One of potential methodologies to minimize the thermal effect of MRF application systems is to optimize the location of the magnetic circuits while considering both the temperature distribution and the distribution of heat generation. In addition, it is known that there are several trade-off behaviors between the temperature effect and application performance. For example, the addition of nanoparticles and proper additives can mitigate the temperature effect, but the field-dependent damper force of the MR damper is decreased.

**Figure 1:**
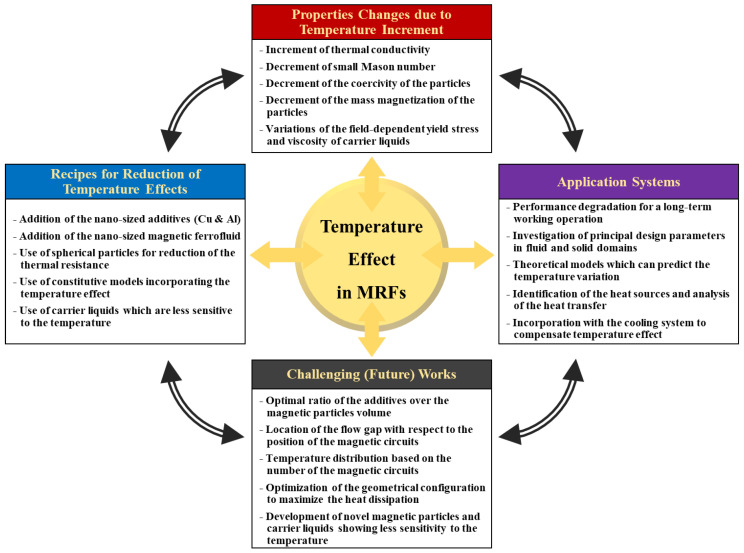
Temperature effects in MRFs and application systems.

The main results on the temperature effect reviewed in Section 4 and Section 5 are summarized and shown in Figure 1. It is clearly seen that the inherent properties of MRFs are weakened due to the temperature increment, and hence, several studies have been conducted to reduce the temperature effect. For example, the addition of nano-sized particles and additives and the use of carrier liquids that are less sensitive to temperature can reduce the property degradation caused by the temperature increment. The temperature problem directly affects the dynamic or control performances of many MRF application systems. Thus, currently, several works are being actively conducted on the formulation of the constitutive models of MRFs while considering the temperature effect. Such models are effective if used from the beginning of the design stage to compensate the temperature effect. Despite many works on the temperature and thermal effect in MRF technology, there are many factors that need to be resolved for successful realization in practical fields. As seen in the list of challenging works in Figure 1, there are several studies that need to be conducted in future. One of challenging works is to find the optimal location of the flow gap, in which the MRF flows in a narrow gap, and the magnetic circuit cores causing the temperature increment from the heat generation. Therefore, the thermal effect of application systems can be significantly reduced through an analysis of the heat transfer of the flow path associated with the magnetic distribution.

## 6. Concluding Remarks

In this paper, review articles about MRFs and their application systems were revisited and summarized in a chronological manner to comprehensively figure out the state of the literature regarding MRF technology. Then, as a major technical contribution, many studies on the temperature and thermal conductivity of MRFs and the heat generation of MRF applications were investigated, and the principal results were summarized, which were mostly achieved over the past two decades. It was identified from the research survey that, even though many different methods to reduce the temperature effect of MRFs have been carried out, there are still issues to be resolved, for example, the optimal ratio of the additives over the particle volume, the proper ratio of the nano-sized particles over the micro-sized particles, the optimal flow gap based on the magnetic circuits, and so forth. As for the application systems, even though many devices and systems have been proposed and tested at a laboratory level, there are impediments to solve for successful realization in a real-world environment, for example, the number of magnetic circuit cores, the analysis of the heat transfer, the optimal geometry considering the diameter and length, the hybrid design with different materials, and so on. In the review investigation on the temperature effect, the formulation of the accurate model of temperature rise due to the magnetic coils driven by the input current, the analysis of the relationship between the semi-active system (energy dissipation only) and temperature variation, the electrical analysis using a power circuit consisting of a resistor and capacitor, and the analysis of the temperature distribution of application system have been discussed in detail.

It should be noted that several review articles on MRFs and their application systems have not been cited in this paper, since the majority of the content of those articles is similar to the review articles cited in this work. Moreover, several studies on the temperature effect of MRFs and their application systems have been investigated, but not cited in this article to avoid the redundancy of the research contents. Finally, it is noted that this review article is not an introduction to MRFs, and, hence, the inherent characteristics, such as field-dependent particle motion and yield shear stress, are not presented as figures. However, interested readers of this review article can refer to any of the review articles cited in this work to understand the salient properties of MRFs and their application systems. The development history of advanced MRFs, their various application systems, the temperature effect on MRFs, and the thermal effect on application systems are given using the tables, respectively.

## Figures and Tables

**Table 1:** Summary of review articles for MRFs.

Years	Main Reviews	Related References
**1** **999**	N/A	
**2000–2009**	(1) Introduction of commercial MRFs made by Lord Company and the verification of a maximum yield stress of 23 kPa at 200 kA/m and a low viscosity without a magnetic field. (2) Development of high performance MR fluids, with low coercivity, a high saturation magnetization, fast response times, spherical-shaped particles, and high purity particles (CIP)	[4,5]
**2010–2019**	(1) Optimization of CIP-based MRFs to achieve minimum off-state viscosity and maximum field-dependent yield stress. (2) Comprehensive revisit of MRFs focusing on the operation mode, yield stress, viscoelastic behavior, particle sedimentation, and constitutive models (3) Methods to enhance sedimentation stability, considering particle size, particle shape, modification of carrier liquids, and a trade-off between sedimentation and MR effect	[6,7,8]
**2020–Present**	(1) **Particle Sedimentation**: Sedimentation stability of MRFs, considering particle size and shape, particle coating, modification of the carrier liquids, and the use of additives. In the particles, the use of smaller, flake-shaped, and coated particles improved the sedimentation stability, and polyalphaolefin (PAO) and 1-hexyl-3-methylimidazolium chloride carrier liquids improved it. Several additives, such as thixotropic agent, carboxylate soap, antioxidant, metal-oxide powders, sulfur-containing, xanthan gum, and stearate carboxylic acid, enhanced the sedimentation stability. (2) **Constitutive Model**: Diverse constitutive models for MRFs, including the Bingham model, biplastic model, Casson model, biviscous model, Herschel–Bulkley (H–B) model, Eyring model, Robertson model, Pa–Casson model, and the Mizrahi–Berk (M–B) model, as well as artificial neural network (ANN) and support vector regression (SVR) techniques, were discussed. Microscopic models, namely, the finite element model, micro–macro model, and the dipole model-based micro–macro model, were also discussed. (3) **Additives**: Influence of modified additives such as carbon and chromium-based additives, and combination of plasticizer–MWCNT–carbon black on the rheological properties of MRFs enhanced the stability of CIPs in the dispersion media.	[9,10,11,12,13,14]

**Table 2:** Summary of review articles of MRF applications.

Years	Main Reviews	Related References
**1999**	N/A	
**2000–2009**	N/A	
**2010–2019**	(1) **Rotary MR Damper**: Characteristics of rotary MR dampers, focusing on their advantages over traditional linear MR dampers were discussed: design compactness, reduced weight, small amount of MRF, and the easiness of hybrid types of MR brakes and clutches. (2) **Sensor Applications**: Sensor applications of MRFs, instead of actuator applications, were introduced: resonant sensors, current sensors, magnetic flux sensors, variable resistors, and tactile sensors for robot surgery. (3) **MR Mount**: Design concepts for a high loaded MR mount were provided by addressing the design process in terms of flow gap, cross section, maximum damping force, distribution of the mount pressure, and avoidance of block-up phenomenon. (4) **Energy Harvesting MR Damper**: A self-powered (energy harvesting) MR damper was introduced, incorporated with an electromagnetic induction (EMI) which generates voltage, and hence power, to operate the MR damper. (5) **Control Strategies**: Control strategies for MR application devices and systems featuring semi-active control were discussed: skyhook controllers, PID controllers, LQR/LQG controllers, sliding mode controllers, adaptive fuzzy controllers, neural network controllers, and hybrid controllers that combine more than two different control schemes. (6) **Surface Polishing**: Surface polishing methods using MRFs were investigated, focusing on polishing mechanisms such as the abrasive particles, held by chains of iron particles, and the bunch of iron and abrasive particles in the form of a micro-chip from the work piece. (7) **Diverse Applications**: Numerous applications were introduced: MR dampers for automotive suspension systems, civil structures, bridge cables, and high story buildings; robotic systems such as deformable grippers; and rehabilitation devices such as prosthetics. (8) **Magnetic Circuit**: The effect of the magnetic circuit position and flow gap direction on the MR effect were discussed, considering the viscosity in the off-state, the length of the flow path, a reduced energy consumption, and a lower manufacturing cost.	[15,16,17,18,19,20,21,22,23,24,25,26]
**2020–present**	(1) **History of MRF Polishing**: The development history of MRF polishing technology from 2016 to 2020 was introduced by addressing its benefits, including recycled MRF, real-time updated abrasiveness, reduced tool wear, stable removal function, and the choice of operation modes, and its drawbacks, such as accurate polishing and slurry polishing. (2) **Power Consumption**: Comparative reports on the MRF operation modes of the flow, shear, and squeeze modes were introduced, focusing on the energy consumption using a new class of magnetic field-sensitive electrolytes, safety, thermal conductivity, and energy storage. (3) **Medical Applications**: The applications of MRF to medical areas and rehabilitation fields were summarized; these include lower limb prosthesis, exoskeletons, orthosis, rehabilitation devices, haptic master, and tactile displays. The studies on the haptic master sensing the resistance force, which is the same with that sensed by the robot, were also reviewed as a possible interface in the telerobotic surgery system. (4) **Large MR Damper**: Large-sized MR dampers were summarized in terms of damping force capacity and design types. In general, a high damping force over 600kN is required for vibration control in civil structures such as buildings and bridge systems. It is suggested that a double-ended MR damper, which contains a gas chamber, is better than a single-ended MR damper for achieving a high damping force. (5) **Metamaterials**: One short review treated MRF as one of several auxetic metamaterials, which have the capability to control their properties and, therefore, are applicable to several dynamic systems. Only the subject of MR prosthetics was considered at this stage, in terms of the negative Poisson’s ratio effect and other relevant properties of metamaterials.	[27,28,29,30,31,32,33]

**Table 3:** Studies on temperature and thermal conductivity in MRFs.

Years	Summary of Main Results	Related References
**~1999**	N/A	
**2000~2009**	(1) **Arrhenius Relationship**: There are many studies on the temperature effect of MRF properties, and one salient property is that the Arrhenius relationship, in which the fluid viscosity is a function of the shear rate, magnetic field, and the temperature, is applicable to predict the temperature dependence via the measurement of the matrix oil. (2) **Mason Number**: It has been studied that the Mason number and Brownian motion have a relationship with the thermal conductivity of MRFs. A small Mason number results in a larger effective thermal conductivity than a large Mason number, and the saturation magnetization of the ferromagnetic material is a monotonically decreasing function of the temperature at a value below the Curie temperature. It has also been found that the Brownian suspension theory can be applicable to explain the temperature-dependent behavior of the carrier liquid.	[35,36]
**2010~2019**	(1) **Thermal Conductivity**: Many works have demonstrated that the thermal conductivity of MRFs is increased by increasing the temperature and particle volume fraction. The heat transfer coefficient is high for a higher particle concentration, and the percentage increase is more pronounced for lower particle concentrations. It has also been shown that the thermal conductivity reaches a maximum stable value when the viscosity continuously grows. (2) **Particle Magnetization**: It has been shown that both the mass magnetization and coercivity of MR particles decrease as the temperature increases, and the phenomenon is particularly obvious at high temperatures. It was also identified that a carrier fluid with a higher viscosity is more sensitive to the temperature variation, and hence, a carrier fluid with low activation energy is required to improve the thermal stability of MRFs in a practical environment. (3) **Nanoparticles**: From the specific MRF investigation, in a nano-sized magnetic particle-based ferrofluid (NMPFF), which has several inherent rheological properties such as improved shear thinning, elevated dynamic moduli, and thermo-rheological complexity, it has been shown from the amplitude sweep test that the crossover strain value increases with the augmenting temperature at a low field, and it decreases with the increasing magnetic field strength. (4) **Additives**: It has been studied that the addition of nano-sized additives such as Cu and Al can enhance the thermal conductivity. It was also identified that the addition of Cu provided a higher thermal conductivity than the addition of Al. As a specific sample, the most prominent increment of the thermal conductivity occurred with the MRF-Cu samples from 35–40 vol% of CIP concentration. (5) **Compressibility Test**: The effect of the temperature on the performance of the compressibility of the MRF has been studied, and it was shown that the shear yield stress of the MRF remains unchanged within the testing range while both the plastic viscosity and the bulk modulus of the MRF decrease as the temperature increases (25 °C~70 °C). It has also been verified that the thermal conductivity of the MRF is also a controllable property, and hence, this can increase or decrease based on the direction of the magnetic field, particle size, and volume fraction of dispersed particles. This behavior was visualized from the observation of the cluster formation at the field-on state, showing that when more and longer chains of particles are formed, the variation in the thermal conductivity of MR fluid is more considerable.	[37,38,39,40,41,42,43,44,45,46,47,48,49,50,51,52,53,54,55,56]
**2020~present**	(1) **Non-Spherical Particles**: The temperature effect of the MRF made of the transformer oil and Fe_3_O_4_ particles has been studied. The thermal increment is higher than the normal spherical-shaped iron-particle-based MRFs due to the shape effect as well as the reduction in the thermal resistance, where a higher particle–particle interaction compared to spherical particles occurs. (2) **Linear Viscoelastic Behavior**: The influence of the temperature on the linear and nonlinear viscoelastic behaviors of MRFs were studied over the temperature range from −5 to 50 °C. Two significant results were found from this work: (i) The MRF is highly dependent on the temperature and shear stress increases when the temperature is reduced, especially below 10 °C, and applying the magnetic field decreases the effect of the carrier fluid, and consequently, the dependency of the MRF on the temperature decreases. (ii) It has been shown that in the linear region, the temperature mostly affects the storage and loss moduli in the absence of the applied flux density, and the critical strain amplitude and associated linear storage modulus are functions of the temperature and applied flux density. (3) **Constitutive Models**: The temperature effect on the properties of MRFs has been analyzed with different constitutive models, conserving the temperature rising from 20 °C to 70 °C. It has been firstly shown that the Herschel–Bulkley model, which has a high accuracy at room temperature, is no longer able to accurately describe the shear stress of MR fluids at high temperatures, causing an error of up to 21.4% at 70 °C. This is because the decrease in shear stress caused by temperature rises is mainly due to the change in the hydrodynamic force between the particles and base carrier fluid, which follows the Navier–Stokes law, which cannot be covered by the Herschel–Bulkley model. Thus, a new constitutive model with temperature prediction was formulated and tested, showing an error of 90% at different temperatures and magnetic field strengths, exhibiting excellent accuracy. (4) **Different Test**: Many testing methods for the temperature effects have been carried out from the different tests: (i) Temperature-dependent yield stress test—the greater the thermal conductivity of the winding, the stronger the thermal conductivity to the surrounding material, and the lower the maximum temperature of the testing device. (ii) Temperature measurement in the damper using an embedded thermocouple—the fluid temperature rises significantly from the atmospheric temperature of 20 °C to 80 °C and 125.39 °C, with a decrease in the damping force by 66.32% at higher loading parameters. (iii) An analysis between the sedimentation and temperature-dependent viscosity—the sedimentation and the viscosity are exponentially reduced as the temperature increases. (iv) Introduction of temperature effect of commercial products—oil and silicone oil-based fluids can typically operate at temperatures from −40 to 150 °C, and water-based fluids are rated from 0 to 70 °C.	[57,58,59,60,61,62]

**Table 4:** Studies on thermal behaviors in MRF applications.

Years	Summary of Main Results	Related References
**~1999**	The heat generation and dissipation of MR damper was studied, and a theoretical model was developed, which could predict the temperature variation of MR damper. It has been shown that the result achieved from the developed non-dimensional model agrees well with the experimental result obtained by assessing the physical parameters.	[63]
**2000~2009**	The performance of the MR damper, including the damper stiffness, was investigated in the temperature range from 15 °C to 75 °C. It was shown that a 34% drop in viscosity, a 22% reduction in yield stress, and a 300% increase in damper stiffness occurred with the rising temperature under certain conditions.	[64,65]
**2010~2019**	(1) **Performance Effects of MR Damper**: Many studies on this issue have been carried out, and the main results are summarized as follows: (i) It was identified that the heat is transferred in both the solid and the fluid domains and it is also generated from the self-inductance of the electromagnetic coil with the fast-changing current and friction of the relative motion between the piston and the cylinder. (ii) It was shown that fluid viscosity has a direct relation with temperature, so MRF viscosity is affected by the dampers’ performance. (iii) In traditional damper design configuration, the temperature appears to be exponentially rising in the flow gap at the beginning, but it slows down when the maximum temperature reaches a certain temperature due to the low thermal conductivity of MR fluid. (2) **Mitigation of Temperature Effect**: There are many recipes to reduce the temperature effect using several different methods. The major methods used to mitigate the adverse effect due to the temperature increment are summarized as follows: (i) The location of the magnetic circuit to minimize the heat generation. (ii) The addition of a proper amount of nanoparticles to achieve the smallest temperature increment. (iii) The development of constitutive models, which can provide the relationship between the temperature effect and energy dissipation.	[66,67,68,69,70,71,72,73,74,75,76,77,78,79]
**2020~present**	(1) **Transient Temperature Distribution**: In most MRF applications, the transient temperature is dominant over the steady state condition. Thus, recently, a few studies on the transient temperature behaviors of MRF application systems were conducted. (i) The thermal problem of the tribological properties of the MR brake was studied by formulating a theoretical analysis of the heating and heat dissipation, demonstrating that the effectiveness of the model is well matched with the measured results at several heating and wear tests, and also showing that the working gap directly correlated with the MRF is the most crucial factor causing performance degradation. (ii) A theoretical model based on the Bingham constitutive model and the temperature-dependent viscosity characteristics was also developed and applied to MR torsional vibration damper with dual heat source. The proposed model was proven via a comparison with the experiment, and it was also demonstrated that the temperature increment at the working domain is the fastest, and the gradual slowing of the temperature rise rate and the maximum temperature of each working condition could be identified from the relationship between the current and temperature. (2) **Special Application Systems**: In general, the working conditions span a wide range of temperatures from −30 °C to approximately 140 °C in the automotive field, but a few existing works have undertaken temperatures below −0 °C 10. However, some special applications using MRFs need to be tested in a wider temperature range. In addition, a durability related to a long-term working operation is crucial to guarantee good performance regardless of the temperature. The following are some special applications of MRFs that have been introduced: (i) the heat generation of two special dampers installed in the naval gun; (ii) large-sized military vehicles subjected to hard driving conditions; (iii) the heat dissipation of high-power MR transmission device operated with wall slip; (iv) a connecting system of several MR dampers for installed ocean platform, which is a requirement of a new cooling structure that is rapidly operated, in which the negative effects of centrifugal force and temperature increment on heat dissipation are observed; (v) a special MR damper in which an aluminum foil bubble insulation material with low thermal conductivity is used in the cavity between the electromagnetic coil and the MRF (call it the heating insulation device); and (vi) a concept of a hybrid damper using more than two devices to reduce the temperature effect, including the brake system, which uses both the MRF and shape memory alloy (SMA), compensating for each other’s weaknesses, and both the stability of the transient fluid dynamics and thermal robustness are improved.	[80,81,82,83,84,85,86]
